# Hospital Extravagance and Expenditure

**Published:** 1888-12-22

**Authors:** 


### HOSPITAL EXTRAVAGANCE AND EXPENDITURE.

A MEETING of the members of The Hospitals Association was held on Wednesday, the 12th inst., at Guy's Hospital, in order to hear a paper read by Mr. P. Michelli, of the Seamen's Hospital Society, and late secretary to St. Mary's Hospital, on "Hospital Extravagance and Expenditure." Lord Charles Bruce was to have presided, but Mr. L. Broke Willoughby, the secretary of the Association, read a letter from his lordship, regretting that his doctor had ordered him not to leave the house in consequence of a bronchial attack. In his absence, Mr. Lushington, the treasurer of Guy's, presided, and briefly introduced Mr. Michelli.

### MR. MICHELLI'S PAPER.

It is a great pity that anybody should need to discuss the question of hospital extravagance. If the burden must of necessity be laid upon some person, it would have been difficult to find a more suitable man than Mr. Michelli. He accomplished his ungracious task exceedingly well at Guy's last week, making many friends and alienating none. Very few wholesome-hearted men love the *rôle* of accusers. It is much pleasanter to praise than to blame. Mr. Michelli blamed so gently—coated his pills with so much of the sweetness of experienced sympathy—that the most hardened offender in the army of wastefulness could not but shed a few repentant tears. For, look at the question as we will, there is no denying the fact that there is money wasted in the hospital world, money which many who subscribe cannot afford to have spent wastefully.

A good many different opinions were expressed about "extras." If there were only one hospital in London, and that were so wealthily endowed as always to close its financial year with a balance at the bank, these extras might be freely allowed, even at the discretion of the house physician and the tender-hearted sister. But sisters and house physicians must try to sympathise with the givers of charity as well as the receivers. Many persons who give to hospitals have to be very sparing at home with costly extras when members of their own families are sick. There should be one rule, and one only, on this point: all extras must be ordered by the visiting physician, and none should be given except in obedience to his instructions.

Costly instruments are probably delicate ground to tread upon, but here, also, Mr. Michelli trod carefully, and undoubtedly felt his way to sound conclusions. Physicians and surgeons ought not to ask for these expensive articles except when they are really needed for the work of the hospital. It is not, perhaps, necessary to say very much on this point, but one thing we must insist upon, viz., that doctors shall spend the money of the hospitals they serve as cautiously as they spend their own. If there is to be any preference, honourable surgeons will see that it is in favour of the exchequer of the hospital. A low tone of professional honour in dealing with charitable funds is not the mark of man of high character and cultivated intelligence.

But the real *piece de resistance* was found when the question of the methods of raising money was reached. It is a scandal—it is a deep disgrace—that some so-called hospitals should spend nearly half their gross income in bringing in the "ways and means." Undoubtedly, the evils of this system are great, and tend to increase. Fortunately, no instance of this kind of recklessness can be adduced against any hospital of standing or position. This is the one fact which the public must bear in mind always, and then they will soon empower their representatives to put an end to the small and unworthy class of institutions which adopt the practice.

Mr. Michelli's remedies are very simple in principle, and would be effective in practice. An identical method of book-keeping for all hospitals, an annual audit by a competent and trustworthy outside authority, and the publication of an annual blue-book would work great changes, probably, indeed, all that are necessary in the management of existing hospitals. The plan is so simple, and might be so easily carried out without serious dislocations of any kind, that we shall be curious to see how the suggestion is received at the different hospitals. Those who really wish to follow a counsel of moderate perfection will put themselves in the way of carrying out the suggestion without delay. We believe the secretary of The Hospitals Association is in communication with some of the principal London treasurers and managers with the view of forming a committee to deal with the subject at once. The question of licensing all new hospitals in order to qualify them to be and to beg is rather a delicate one, and will require a good deal of consideration.

The debate which followed the reading of Mr. Michelli's paper was well sustained throughout, as will be seen from the following report. Those who have not seen this paper should apply to Mr. Broke Willoughby, at The Hospitals Association, Norfolk House, Norfolk Street, Strand, for copies.

The Discussion.

Mr. Malcolm Morris (St. Mary's Hospital) opened the discussion, paid a tribute to Mr. Michelli for the skill and tact with which he had introduced his subject, and remarked that the paper had three great good qualities, inasmuch as it was short, concise, and concrete. As far as the second part of the paper was concerned, he entirely agreed with every word, and he emphasised the difficulty of understanding how the hospitals were conducting their business in the absence of an uniform method of presenting accounts. The facts in connexion with unendowed hospitals, supported by public money as they were, ought to be common property, for the public had a right to know what was done with their funds. Uniformity of accounts would assist the work of hospitals amazingly. The suggestion that The Hospitals Association should endeavour to obtain a Government audit of hospital accounts was an excellent one, for the present system of auditing was a mere farce. He himself had actually audited the accounts of his own hospital. As to licences, he considered hospitals should be required to have a licence from Government as much as a public-house, because some hospitals were calculated to do as much harm as public-houses were. At present anybody could put a board outside a private house and say, "This is a special hospital." Before such a thing was allowed, it ought to be distinctly understood that such an institution was really required for the use of the suffering poor of the neighbourhood. As to the direct extravagance, he considered Mr. Michelli was a little severe in his remarks as to the rarity of physicians and surgeons possessing a great knowledge of business, for many men took pains to try and become business men for the simple reason that it was so exceptionally difficult to get business men to come and take charge of hospitals, and The Hospitals Association had a great future work before it in endeavouring to influence business men to come and take part in the management of hospitals. At present there were far too few business men taking part in hospital work, and the more light which was thrown into the hospitals and the more people who could be got to come into the hospitals and attend meetings, and take a real interest in the work the better it would be for the institutions themselves, and the better it would be for the public. He considered that the points of direct extravagance to which Mr. Michelli had referred were not so serious after all, and that the trifling matters of eggs and grapes had been unnecessarily enlarged on. The speaker asked how Mr. Michelli had obtained his figures in respect to the cost of advertising per occupied bed when he stated in his paper that in looking through the reports there was nothing to tell which hospital spent five shillings and which spent twenty times five. In conclusion, he expressed a desire to see a Royal Commission appointed to inquire into special hospitals, and congratulated The Hospitals Association upon such a practical paper.

Mr. Burdett remarked upon the courage of the lecturer in dealing with his subject, and proceeded to say that his experience in seeking for information from the authorities of hospitals was that those authorities were anxious to give all the information in their power to elucidate any difficult point upon the application of any person who had a reasonable right to seek the information. The reason able right was judged by the interest the inquirer took in hospital questions. He had never had any difficulty, and had never met with a refusal, finding, upon the contrary, that the committees were most anxious to co-operate with one in trying to show how institutions stood relatively to one another. A great difficulty in the way of collating information was the absence of any recognised form of accounts, and Mr. Burdett pointed to the railways of this country as an example where Parliament took the matter up and formulated a system which now constituted the basis upon which every railway had to keep its accounts. He should like to see some initiatory step taken by the managers of the leading hospitals to have a form of accounts printed and widely circulated, and he believed that if this were done, the form would be cordially welcomed and very generally adopted. As to the necessity in hospital accounts of always showing a deficiency, the speaker, as an old beggar, admitted this axiom of hospital management, pointing out that in the absence of a deficiency, some of the supporters of the hospital believed that the charity was in funds, and gave their money for one year in another direction —a very serious matter for the first charity. He therefore considered that it was right for a hospital to show a deficiency if this was possible. There was an amusing side to this deficiency question, which had become such a usual practice that the auditors of the accounts of the Pay Hospital—one of the largest firms of accountants in the City of London—on one occasion actually manufactured a deficiency, although, as a matter of fact, there was an amount of some thousands of pounds at the time to the credit of the association. The speaker proceeded to refer to recent correspondence in the newspapers on a suggestion that the Charity Organisation Society should obtain particulars of all hospitals, so that everybody would be able to take the society's list and see which was a deserving charity and which was an undeserving one. The secretary declined the honour and labour, but suggested that all hospitals should be placed under the registrar of friendly societies, which would result in a common basis and common form of account. Then the registrar of friendly societies wrote that there would be no objection to such a course, and added, as a point of interest, that some time before the Charity Organisation Society had raised this point, and it was then suggested to them that as they were a voluntary organisation, it would be very proper for them to make an example by themselves coming under the Friendly Societies' Act and rendering accounts. This the Charity Organisation Society unanimously rejected, and it was evident, as far as these gentlemen were concerned, that what was sauce for the goose was not sauce for the gander. Mr. Burdett warmly expressed his opinion that the representatives of the public should have a right to say legally whether any proposed new hospital was wanted or not. In the absence of such a system semi-bogus and useless charities had often sprung up, and would continue to spring up, diverting the funds from much-needed and rightful objects, and early legislation should be insisted upon. At any rate, the Local Government Board should be moved, in order to secure that the new County Councils had the power to regulate these matters. As to the payment of commission, he heard of a case years ago of a special hospital where £3,000 was raised by voluntary contributions, and £2,000 of it was said to have been spent in commission and advertisements. As to Mr. Morris's question, Mr. Michelli evidently meant that there was usually nothing on the face of the reports of many hospitals to show how much was spent on advertisements. If, however, the accounts were analysed, and that analysis was supplemented by a letter asking a few questions, it was possible to get the exact figures, and that was probably what Mr. Michelli had done. The tables would have been improved if the number of beds and type of hospital were given, so that judgment might be formed as to whether the hospitals were large or small. In conclusion, the speaker said that after ten months' work a complete analysis of the whole of the accounts of the chief hospitals of England, Scotland and Ireland had been compiled, which would give full particulars of every hospital. These figures would be published in the *Hospital Annual*, and those who wanted to distribute their money wisely would have in that work abundant proof of the needs of the really deserving hospitals.

Mr. Ryan (St. Mary's Hospital) congratulated the lecturer on the way in which he had pointed out what was in his mind, and had dealt with somewhat delicate matters. It was to the multiplication of small details that the public had to look for the cause of some of the greatest extravagances in public hospitals. The figures as to the cost of advertising struck him with astonishment, and he thought it would be extremely valuable if Mr. Michelli would state what the classes of hospitals were to which he referred. Most probably he did not refer to general hospitals, and he hoped it would not go forth to the public that this scandalous expenditure attached to the large general hospitals of the metropolis. He questioned the value of statistics as to the cost of a hospital bed in gauging the economy or extravagance of an institution, and quoting figures in connexion with the St. Mary's Hospital and the Manchester Royal Infirmary, which last he said was the very tip top apex of economy, stated that on the question of provisions, the cost per unit throughout the whole range of provisions at St. Mary's, taking the whole at 100, was not more than 87 of the Manchester Royal Infirmary. On gas there was a difference of some 17 per cent., and on coal there was a tremendous difference, Manchester paying 8s. 7¾d. a ton, and St. Mary's 15s. 4d. per ton. These figures showed that the cost per occupied bed must be very carefully corrected before it was of any value as a standard of economy or extravagance. Even where the same articles were supplied at the same price, the varying circumstances in the different institutions had to be considered. But when all the necessary corrections were made, the London hospitals, it would be found, cost more than the provincial hospitals. Mr. Ryan commented upon the interference of persons who did not understand the administration of hospitals with the officers who did, and said that government by individuals whose sole qualification was a money payment to the institution was little short of a scandal.

Surgeon-Major Ince agreed, almost without exception, to the remarks which had been made in the paper, and during the discussion. There was no doubt in the world that a great deal of most unjustifiable extravagance went on, and there was a great necessity for reform in the management of our hospitals. There was no extravagance or mismanagement in the hospitals and dispensaries in India, because they were under Government administration. He scarcely considered that the giving of extras in the shape of grapes, eggs, &c., was a very heinous offence, and remarked that the young house-surgeon now was very liable to be tempted to allow these little delicacies, at the request of the present class of nurses, whose physical and intellectual qualities were so great an improvement over those of the nurses of previous years. There was much extravagance in the matter of alcohol, and though it had been suggested that the point was a moot question, he claimed that it was a settled question, and that it had been proved by the establishment of a hospital on non-alcoholic principles that there was no necessity whatever for the administration of alcohol. The real remedy for all the evils which had been spoken of that night was a more strict supervision of those who were called upon to administer the funds of charity.

Dr. Potter first referred to the high figures which were seen in one of the short tables read by Mr. Michelli, being extracted from the reports of certain hospitals. In thanking Mr. Michelli for his admirable paper, he said he was himself a Gorgon for economy, and was constantly getting into trouble with his secretary. He feared the paper would fall dead, and that no more would be heard of it, for want of some practical means of bringing pressure upon the hospitals, so that a uniform system of accounts could be adopted, and everybody be his own auditor.

A Gentleman in the meeting, whose name did not transpire, and who was very indistinctly heard, maintained that the charges of extravagance which had been made did not coincide with the experience of hospitals which he had had.

Dr. Steele proposed a vote of thanks to Mr. Michelli for his excellent paper, and remarked that the subject could not be a popular one with hospitals, for all the institutions prided themselves upon their economy. When, however, there was such a variety in the cost per bed, one was bound to ask the meaning of it. The public at large, however, did not look very closely into these matters, and were only anxious to know that the patients in the hospitals were well fed and well doctored. It was a thousand pities that there was not a standard by which a good hospital and a bad could be judged. There was, as the meeting appeared to feel, a great want of uniformity in regard to hospitals, which ought to be remedied in some way. For instance, there was the variation in the cost of out-patients, extending from 6d. to 15s. and 20s. A hospital associated with a medical school must be more expensive than a hospital not so associated. Dr. Steele drew attention to the iniquitous taxes which he said had been imposed upon hospitals for the last twenty years, and remarked that this increased the cost of the beds £4 or £5 each. Steps were being taken to endeavour to obtain a restoration of the exemption, and he hoped the various hospitals would support the Bill to the utmost possible extent. He agreed as to the necessity and propriety and expediency of having some sort of supervision over the hospitals in the shape of some central board, and suggested that the Charity Commissioners should examine into the accounts of the hospitals. Some authority which could prevent the formation of the numerous special hospitals which were not wanted was absolutely necessary in the interests of the general hospitals at large.

The Chairman agreed with the difficulties in the way of ascertaining the relative cost of large hospitals, and suggested that the Local Government Board or the County Council should be pressed to introduce some system for the preparation of accounts. There was much in the argument as to the varying cost of varying hospitals, but he hoped the meeting would pass a vote of thanks to Mr. Michelli, and would further resolve that the paper was worthy of the careful consideration of The Hospitals Association, in order that the Association might formulate either a letter or a petition to the authorities, with a view to procure some one form of account which would be acceptable to all the world. Both these suggestions were adopted, Mr. Nixon stating that he cordially supported the idea of a general system of accounts.

